# Challenges and Strategies in Administering Anesthesia to Pregnant Patients With Malaria: A Comprehensive Review

**DOI:** 10.7759/cureus.67285

**Published:** 2024-08-20

**Authors:** Renuka Patond, Nikhil Bhalerao

**Affiliations:** 1 Department of Anesthesiology, Jawaharlal Nehru Medical College, Datta Meghe Institute of Higher Education and Research, Wardha, IND

**Keywords:** hemodynamic stability, anesthetic management, maternal anemia, anesthesia, pregnancy, malaria

## Abstract

Malaria remains a significant global health challenge, particularly in sub-Saharan Africa, where its impact on pregnant women and their fetuses is profound. The disease's complex interaction with pregnancy introduces unique challenges in anesthesia management, necessitating a thorough understanding of both malaria and its implications for anesthetic care. This review aims to explore the multifaceted issues associated with anesthesia for pregnant patients with malaria, examining the impact of the disease on pregnancy and the specific considerations required for effective anesthetic management. A comprehensive review of the current literature was conducted, focusing on the physiological effects of malaria on pregnancy, its complications, and the related anesthetic challenges. The review synthesizes findings from clinical studies, case reports, and expert guidelines to provide an overview of best practices and strategies. Malaria in pregnant women can lead to severe complications such as maternal anemia, placental insufficiency, and preterm labor, all of which complicate anesthetic management. The review identifies key considerations for anesthesia, including the choice of anesthetic techniques, drug interactions, and fluid management. Specific challenges include managing anemia, ensuring adequate hemodynamic stability, and mitigating potential risks associated with malaria medications. Effective anesthesia management in pregnant patients with malaria requires a nuanced approach that addresses both the disease's effects and the physiological changes of pregnancy. This review underscores the need for tailored anesthetic strategies and highlights areas for further research to enhance patient safety and outcomes. Recommendations are provided to guide clinicians in optimizing care for this vulnerable population.

## Introduction and background

Malaria is a life-threatening disease caused by protozoan parasites of the genus Plasmodium, transmitted through the bite of infected female Anopheles mosquitoes [1]. Despite significant progress in malaria control and prevention, the disease remains a major global health issue [2]. According to the World Health Organization, malaria continues to affect over 200 million people annually, with sub-Saharan Africa being the most heavily impacted region. The disease is characterized by recurring fever, chills, and flu-like symptoms, which can escalate to severe complications if left untreated [3]. Pregnant women are particularly vulnerable to malaria due to physiological changes that alter immune responses and increase susceptibility to infections. Malaria during pregnancy can lead to severe complications, including maternal anemia, placental malaria, and preterm delivery [4]. Maternal anemia, a common consequence of malaria, can exacerbate the risk of adverse outcomes for both the mother and the fetus. Fetal complications include intrauterine growth restriction, low birth weight, and an increased risk of neonatal mortality. The interaction between malaria and pregnancy introduces additional complexities in managing the disease and the associated obstetric conditions [5].

Effective anesthesia management in pregnant patients with malaria is crucial due to the increased risks associated with both the disease and the physiological changes of pregnancy. Anesthesia strategies must account for the potential impact of malaria-related complications such as anemia, altered hemodynamics, and the need for careful fluid management [6]. The choice of anesthetic techniques and agents requires careful consideration to ensure both maternal safety and optimal fetal outcomes. Understanding the interplay between malaria and anesthesia is essential for minimizing risks and enhancing the overall quality of care for these vulnerable patients [7]. This review aims to comprehensively analyze the challenges and strategies associated with providing anesthesia for pregnant patients affected by malaria. By examining the intersection of malaria, pregnancy, and anesthesia, this review highlights current practices, identifies areas needing further research, and proposes evidence-based recommendations to improve patient outcomes. The ultimate goal is to enhance the understanding of anesthesia management in this specific context and contribute to developing more effective and safer clinical practices.

## Review

Pathophysiology of malaria in pregnancy

Malaria during pregnancy poses significant risks to both maternal and fetal health, particularly in endemic regions. Pregnant women are especially susceptible to severe forms of malaria due to physiological changes during pregnancy, such as alterations in the immune response [8]. This heightened susceptibility can lead to severe complications, including maternal anemia, hypoglycemia, and pulmonary edema. The mortality rate from severe malaria in pregnant women can be two to ten times higher than in nonpregnant individuals. These risks are compounded by the substantial morbidity associated with malaria, necessitating careful management and monitoring throughout pregnancy [9]. The effects of malaria extend beyond the mother, significantly impacting fetal health. Infected pregnant women face a higher risk of adverse pregnancy outcomes, including intrauterine growth restriction, preterm delivery, stillbirth, and neonatal death. These adverse outcomes often result from accumulating malaria parasites in the placenta, particularly in infections caused by *Plasmodium falciparum* [4]. The parasites can sequester in the placental intervillous space, leading to inflammation and disruption of normal placental function. This disruption impairs nutrient and oxygen transport to the fetus, contributing to low birth weight and other developmental issues [10]. Malaria-related complications during pregnancy are multifaceted and can have lasting effects on both maternal and fetal health. One of the most common complications is anemia, which arises from the destruction of red blood cells by the malaria parasite, exacerbated by the physiological demands of pregnancy [11]. Additionally, placental malaria can cause histological changes, including immune cell infiltration and malaria pigment deposition, further compromising placental function. These abnormalities not only affect the immediate health of the fetus but also have implications for long-term health outcomes. The risk of stillbirth and low birth weight is significantly elevated in pregnancies complicated by malaria, underscoring the need for effective prevention and treatment strategies [12].

Anesthesia considerations in pregnant patients with malaria

**Figure 1:**
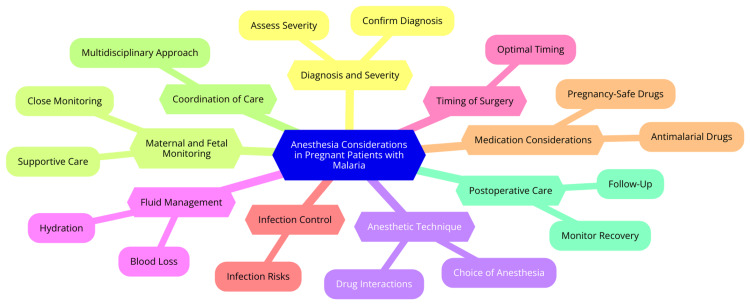
Anesthesia considerations in pregnant patients with malaria Image credit: Renuka Patond

Anesthesia considerations in pregnant patients with malaria are complex due to the interplay of physiological changes during pregnancy, the effects of malaria on anesthetic management, and the specific challenges posed by the disease. Understanding these factors is crucial for ensuring the mother's and fetus's safety and well-being [13]. Pregnancy induces several physiological changes that significantly impact anesthetic management. One notable change is in the cardiovascular system, where increased blood volume and cardiac output alter hemodynamics, necessitating careful monitoring of blood pressure and fluid status during anesthesia [14]. Respiratory changes also occur as the diaphragm elevates and oxygen demand increases, affecting ventilation and requiring adjustments in anesthetic techniques and monitoring. Furthermore, pregnancy is associated with a hypercoagulable state, complicating the management of patients with malaria, particularly those suffering from thrombocytopenia or coagulopathy. Altered drug metabolism is another critical consideration; pregnancy changes the pharmacokinetics of anesthetic agents, often necessitating dosage adjustments due to increased plasma volume and altered hepatic metabolism [15]. Malaria significantly complicates anesthetic management in pregnant patients through various mechanisms. One primary concern is altered drug metabolism resulting from the disease, which can lead to interactions with anesthetic agents. For instance, antimalarial drugs may cause complications like hypoglycemia and QT prolongation, requiring careful monitoring and potential adjustments in anesthetic dosages [16]. Malaria also increases the risk of bleeding due to thrombocytopenia and coagulopathy, heightening the risk during surgical procedures. It is essential to assess platelet counts and coagulation status before proceeding with neuraxial anesthesia, which is generally preferred unless contraindicated. Moreover, the severity of malaria can lead to complications such as cerebral malaria, which may necessitate general anesthesia due to risks associated with airway management and potential increased intracranial pressure (ICP) during procedures [17]. The presence of malaria introduces several specific challenges for anesthetic management. Pregnant patients are particularly susceptible to severe malaria, resulting in complications such as anemia, hypoglycemia, and pulmonary edema. The mortality rate from severe malaria in pregnant women is significantly higher than in nonpregnant populations, underscoring the need for careful monitoring and management [11]. Additionally, malaria can lead to multiple organ dysfunction, complicating the anesthetic approach. Conditions such as renal impairment, respiratory distress, and cardiovascular instability must be closely monitored and managed throughout the perioperative period. Another critical consideration is the potential for vertical transmission of malaria during pregnancy and delivery, which adds complexity to the management of anesthesia in these patients. The risk of fetal distress due to maternal hypoglycemia necessitates vigilant monitoring of blood glucose levels during the perioperative period [16]. Anesthesia considerations in pregnant patients with malaria are shown in Figure 1.

Preoperative assessment and preparation

Preoperative assessment and preparation for pregnant patients with malaria involve several critical components, including diagnostic procedures, optimization strategies, and coordination with healthcare specialists [18]. Diagnostic and screening procedures are essential for evaluating the patient's overall health status and the severity of the malaria infection. A complete blood count assesses anemia and thrombocytopenia, which are common in malaria cases. A blood smear identifies the type of malaria parasite and the degree of parasitemia, providing crucial information about the infection's severity [19]. A coagulation profile checks for disseminated intravascular coagulation (DIC) and helps plan for potential bleeding complications during surgery. Renal function tests measure urea and creatinine levels and evaluate kidney function, which can be impaired in severe malaria. Monitoring electrolytes (including magnesium and calcium) and liver function is vital, as imbalances can occur in severe malaria, impacting fluid management and anesthetic choices. Depending on the clinical scenario, additional tests such as chest X-rays, arterial blood gases, and ECG may be warranted to evaluate respiratory and cardiac function, particularly in severe cases [20]. Preoperative optimization and stabilization strategies aim to reduce systemic effects and improve maternal and fetal outcomes. Prompt and effective treatment of malaria should be initiated before surgery. Careful fluid resuscitation is crucial, especially in patients with signs of dehydration or hypovolemia [21]. Monitoring central venous pressure can guide fluid replacement strategies to avoid pulmonary edema, a common complication in severe malaria. If the hematocrit falls below 25%, blood transfusion may be necessary, but caution is required to avoid fluid overload and subsequent pulmonary complications. Regular checks for hypoglycemia are essential, particularly if the patient is receiving quinine treatment, as maternal hypoglycemia can lead to fetal distress. Evaluating the patient's consciousness level using the Glasgow Coma Scale is important, especially in cases of cerebral malaria, to anticipate postoperative complications [22]. Effective management of pregnant patients with malaria requires a multidisciplinary approach. Coordination with obstetricians is critical to address the unique challenges of pregnancy, including fetal monitoring and managing obstetric complications related to malaria [23]. Collaboration with infectious disease experts ensures appropriate treatment protocols are followed and that the patient receives optimal care for malaria management, particularly in severe cases. Regular communication among the surgical team, obstetricians, and infectious disease specialists is essential for preoperative planning and intraoperative decision-making, ensuring maternal and fetal health is prioritized throughout the surgical process [24].

Anesthetic techniques and management

Managing anesthesia in pregnant patients with malaria presents unique challenges that require careful consideration of the disease's implications. This section discusses the indications and considerations for both general and regional anesthesia and effective pain management strategies [16]. General anesthesia is often indicated in cases of severe malaria, particularly when patients exhibit significant complications. For instance, severe anemia, characterized by critically low hemoglobin levels, may necessitate general anesthesia to ensure adequate airway management and hemodynamic stability [16]. Additionally, thrombocytopenia, where platelet counts fall below the threshold for neuraxial anesthesia, can make general anesthesia safer, avoiding the risks associated with neuraxial techniques. In cases of cerebral malaria, where there is a risk of increased ICP or neurological involvement, general anesthesia allows for better airway control and hemodynamics, especially during emergencies [25]. When administering general anesthesia, careful selection of drugs is crucial due to potential interactions with antimalarial medications. Commonly used induction agents include thiopental and propofol; however, caution is advised with ketamine, as it may increase cerebral blood flow and ICP. Neuromuscular blockers require careful dosage adjustments because quinine can enhance their effects. Continuous monitoring of hemodynamic status and blood glucose levels is essential, as hypoglycemia is a frequent complication associated with malaria [26]. Regional anesthesia techniques, such as spinal and epidural anesthesia, may be suitable in certain circumstances, especially when there is no significant coagulopathy present. If the patient does not exhibit severe thrombocytopenia or coagulopathy, neuraxial anesthesia can be considered safe and effective. In a well-monitored setting, regional anesthesia provides effective pain relief while minimizing the risks associated with general anesthesia [27]. However, significant challenges and contraindications to regional anesthesia must be considered. The risk of coagulopathy is a primary concern; the presence of thrombocytopenia and DIC increases the risk of hemorrhage with neuraxial techniques. Furthermore, while there is no documented evidence of parasite transfer to the cerebrospinal fluid via spinal anesthesia, theoretical risks persist, particularly in patients with suspected cerebral malaria. Fluid management is also critical, as careful monitoring is needed to avoid exacerbating cerebral edema, especially in patients with compromised hemodynamics [28]. Effective pain management in malaria-infected pregnant patients should consider the potential complications associated with the disease. Opioids can be utilized for pain relief; however, their use must be balanced against the risk of respiratory depression, particularly in patients with compromised respiratory function due to malaria. Nonsteroidal anti-inflammatory drugs (NSAIDs) may also be employed cautiously, considering their potential effects on platelet function and renal function in the context of malaria [29]. In addition to pharmacological options, adjunctive therapies can enhance pain management while addressing malaria-related complications. Regional anesthesia can provide effective analgesia when appropriate while minimizing systemic drug exposure. Medications such as acetaminophen can be safely used for mild to moderate pain, but careful monitoring for drug interactions is essential [30].

Intraoperative management

Intraoperative management of pregnant patients with malaria requires a comprehensive approach to address the unique challenges posed by the disease. Close monitoring of malaria-related complications is essential during surgery. Fluid management is particularly critical, as malaria can lead to hypovolemia due to hemolysis and fluid shifts [16]. Anesthesiologists must carefully resuscitate the patient to maintain hemodynamic stability, continuously monitoring vital signs and fluid balance to prevent both hypovolemia and fluid overload. Additionally, attention to cerebral blood flow dynamics is crucial, especially in cases of cerebral malaria, where increased ICP may occur due to cerebral edema. Anesthetic techniques that minimize increases in ICP should be prioritized, and certain drugs that elevate cerebral blood flow should be avoided [16]. Blood product considerations are also vital in the management of these patients. Severe malaria often presents with anemia and thrombocytopenia, necessitating a careful assessment of the need for blood transfusions. The decision to administer packed red blood cells should be based on hemoglobin levels. At the same time, platelet transfusions may be required if counts are critically low, particularly in patients undergoing neuraxial anesthesia. Fluid resuscitation should be approached with caution, using isotonic fluids as a standard and considering colloids in cases of significant hypoproteinemia. Continuous monitoring for signs of fluid overload, especially in patients with pulmonary edema, is essential to prevent adverse outcomes [31]. Minimizing infection risk during surgery is another critical component of intraoperative management. Strict aseptic techniques must be adhered to, and prophylactic antibiotics may be indicated to reduce the risk of secondary infections, particularly in patients undergoing surgical interventions. Effective analgesia is also a priority, and the choice of analgesic technique should consider the patient's coagulation status and the severity of malaria. While neuraxial anesthesia may be appropriate without significant thrombocytopenia or coagulopathy, general anesthesia may be preferred in cases of severe disease or concerns about the patient’s hemodynamic stability [32]. Finally, a multidisciplinary approach is crucial for optimizing care in pregnant patients with malaria. Collaboration among anesthesiologists, obstetricians, and infectious disease specialists ensures that all aspects of the patient’s health, including managing malaria and the surgical procedure, are comprehensively addressed. This teamwork is essential for achieving positive outcomes in this vulnerable population, highlighting the importance of coordinated care in complex clinical scenarios [13].

Postoperative care

Postoperative care for pregnant patients with malaria is crucial to monitor for complications, manage pain, and prevent infection or relapse. Vital signs, including temperature, heart rate, blood pressure, and respiratory rate, should be closely monitored to detect signs of infection or complications. Fluid balance is also essential, with close fluid intake and output monitoring to prevent fluid overload or dehydration. Regularly checking hemoglobin and platelet count is necessary to detect anemia and thrombocytopenia, which can worsen postoperatively. To detect acute kidney injury, renal function should be monitored by assessing urine output and serum creatinine levels. Additionally, neurological status should be evaluated for signs of cerebral malaria, such as altered mental status or seizures [11]. Pain management is a critical aspect of postoperative care. A multimodal approach using a combination of acetaminophen, NSAIDs, and opioids is effective for pain control while minimizing opioid-related side effects. Early mobilization and physiotherapy should be encouraged to prevent complications like deep vein thrombosis and pneumonia. Nutritional support is also essential, ensuring adequate caloric and protein intake for wound healing and recovery [33]. Infection control and malaria management are crucial in the postoperative period. Antimalarial therapy should be continued as prescribed, with dose adjustments if necessary based on the patient's condition. Strict hand hygiene protocols and appropriate personal protective equipment are essential to prevent nosocomial infections. Monitoring for signs of malaria relapse or recrudescence, such as fever, chills, and parasitemia, is crucial, and prompt treatment with antimalarials is necessary. Finally, considering malaria chemoprophylaxis for the postpartum period, especially if the patient resides in or travels to an endemic area, may be beneficial [34].

Case studies and evidence-based recommendations

Recent case studies illustrate the complexities and considerations in administering anesthesia to pregnant patients with malaria. A 2016 case published in the Korean Journal of Anesthesiology documented the successful use of spinal anesthesia in a pregnant patient with acute malaria who required an urgent cesarean delivery. The patient had a platelet count of 90,000/μL but showed no signs of coagulopathy. The spinal anesthesia was performed without complications, and the patient experienced a smooth recovery postoperatively, demonstrating the feasibility of neuraxial techniques in selected cases [35]. In contrast, a 2014 case report in the Indian Journal of Anesthesia described a more challenging scenario involving a 24-year-old pregnant woman at 37 weeks of gestation who was afflicted with cerebral malaria and leptospirosis coinfection. Due to her altered mental status, seizures, and hemodynamic instability, general anesthesia was deemed necessary for her emergency cesarean section. This case underscores the importance of assessing the severity of the patient's condition when determining the appropriate anesthetic approach, as severe systemic issues may preclude using neuraxial techniques [36]. Based on these case studies and recent literature, several evidence-based recommendations can be made for the anesthetic management of pregnant patients with malaria. First, a thorough preoperative assessment is essential, focusing on the type and severity of malaria and any signs of end-organ impairment. Identifying severe infections is crucial for anticipating complications during surgery [35,36]. Regarding anesthesia, spinal anesthesia is often preferred when the patient does not present with severe complications. Its effectiveness and lower risk profile make it suitable for many patients. However, general anesthesia may be necessary if significant systemic issues are present or urgent intervention is required. The decision should be tailored to the patient's presentation [35,36]. Intraoperative management should carefully consider fluid balance and monitoring cerebral blood flow dynamics. Preventing hypoglycemia is particularly important, as antimalarial treatments like quinine can exacerbate maternal hypoglycemia. Continuous monitoring of blood glucose levels is recommended, especially if fetal distress is suspected [35,36]. Postoperative care is critical, especially for patients with severe malaria. Close monitoring in a critical care setting may be required, as these patients could need ongoing renal support, ventilation, or inotropic therapy. It is also advisable to repeat blood films every 8-12 hours until negative results are obtained to monitor for residual parasitemia [16].

Future directions and research

The anesthesia management in pregnant patients with malaria reveals several critical gaps in knowledge and practice, offering significant opportunities for future research and improvement. One of the primary challenges is the limited availability of comprehensive data specific to this population. Much of the existing knowledge stems from case reports and studies conducted in malaria-endemic regions, which may not apply to other settings. This scarcity of large-scale studies impedes the development of evidence-based guidelines and best practices [6]. Moreover, the absence of standardized protocols for anesthetic management in pregnant patients with malaria leads to variability in practice. Clinicians often encounter uncertainty when choosing between neuraxial and general anesthesia, determining fluid management strategies, and setting monitoring protocols. This inconsistency can affect patient outcomes and complicate clinical decision-making. Additionally, there is a lack of understanding regarding the interactions between various antimalarial drugs and anesthetic agents. Specifically, their effects on hemodynamic stability, glucose levels, and coagulation status during the perioperative period are not well-documented [6]. Emerging trends suggest a growing preference for neuraxial anesthesia in this context due to its potential to mitigate risks associated with airway management complications, such as edema. However, further research is needed to confirm its safety and efficacy. Enhanced monitoring techniques, such as continuous glucose monitoring and advanced hemodynamic assessments, could improve patient outcomes by providing real-time data for managing malaria-related challenges. A multidisciplinary approach involving obstetricians, anesthesiologists, and infectious disease specialists can also improve patient care by addressing obstetric and infectious issues [13]. Several areas require further research and clinical trials to address these gaps and improve anesthetic management. First, studies focused on the safety and efficacy of neuraxial anesthesia in pregnant patients with malaria are needed, particularly concerning risks related to coagulopathy and cerebral malaria. Controlled trials could yield valuable insights into best practices. Additionally, investigating the pharmacokinetics and pharmacodynamics of antimalarial drugs about anesthetic agents could clarify their interactions and inform safer management protocols [37]. Longitudinal studies examining maternal and fetal outcomes following various anesthetic techniques in malaria contexts could help establish evidence-based guidelines and explore long-term effects on both mothers and infants. Finally, developing clinical guidelines based on robust evidence could standardize care for pregnant patients with malaria, addressing current inconsistencies and ultimately improving outcomes. By focusing on these areas, the medical community can enhance the safety and effectiveness of anesthetic management for this vulnerable population [38].

## Conclusions

In conclusion, the management of anesthesia in pregnant patients with malaria presents unique and complex challenges that require a nuanced approach. The interplay between malaria's impact on pregnancy and the physiological changes that occur during gestation necessitates careful consideration of anesthetic techniques and agents to mitigate risks and optimize outcomes. Maternal complications such as anemia and hemodynamic alterations, along with the potential impact on fetal well-being, underscore the need for tailored anesthesia strategies. This review highlights the importance of integrating current knowledge with clinical practice to address these challenges effectively. Future research should focus on refining anesthesia protocols, exploring innovative techniques, and improving our understanding of how malaria affects anesthetic management. By advancing our approach to anesthesia in this high-risk population, we can enhance both maternal and fetal safety, ultimately contributing to better health outcomes in the context of malaria-affected pregnancies.

## References

[1] Crutcher JM, Hoffman SL (1996). Medical Microbiology. Medical Microbiology.

[2] Pattanshetty S, Dsouza VS, Shekharappa A (2024). A scoping review on malaria prevention and control intervention in fragile and conflict-affected states (FCAS): a need for renewed focus to enhance international cooperation. J Epidemiol Glob Health.

[3] (2024). Fact sheet about malaria. https://www.who.int/news-room/fact-sheets/detail/malaria.

[4] Rogerson SJ, Mwapasa V, Meshnick SR (2007). Malaria in Pregnancy: Linking Immunity and Pathogenesis to Prevention. Perspectives: Supplement to Volume 77(6) of American Journal of Tropical Medicine and Hygiene. American Society of Tropical Medicine and Hygiene.

[5] Bauserman M, Conroy AL, North K, Patterson J, Bose C, Meshnick S (2019). An overview of malaria in pregnancy. Semin Perinatol.

[6] Khanna A, Dua N, Sehgal R, Sood J (2016). Anaesthetic management of parturient with malaria and thrombocytopaenia. Indian J Anaesth.

[7] Upadya M, Saneesh PJ (2016). Anaesthesia for non-obstetric surgery during pregnancy. Indian J Anaesth.

[8] Unger HW, Acharya S, Arnold L (2023). The effect and control of malaria in pregnancy and lactating women in the Asia-Pacific region. Lancet Glob Health.

[9] Takem EN, D'Alessandro U (2013). Malaria in pregnancy. Mediterr J Hematol Infect Dis.

[10] Seitz J, Morales-Prieto DM, Favaro RR, Schneider H, Markert UR (2019). Molecular principles of intrauterine growth restriction in Plasmodium falciparum infection. Front Endocrinol (Lausanne).

[11] Schantz-Dunn J, Nour NM (2009). Malaria and pregnancy: a global health perspective. Rev Obstet Gynecol.

[12] Zakama AK, Ozarslan N, Gaw SL (2020). Placental malaria. Curr Trop Med Rep.

[13] Zahavi I, Fons M, Meir M, Volevich M, Guasch E, Nunnally M, Einav S (2024). Anesthetic approach to pregnant patients with malaria: a narrative review of the literature. J Anesth Analg Crit Care.

[14] Luthra A, Bajaj R, Jafra A, Jangra K, Arya VK (2017). Anesthesia in pregnancy with heart disease. Saudi J Anaesth.

[15] Brinkman JE, Toro F, Sharma S (2024). Physiology, Respiratory Drive. https://www.ncbi.nlm.nih.gov/books/NBK482414/.

[16] Soltanifar D, Carvalho B, Sultan P (2015). Perioperative considerations of the patient with malaria. Can J Anaesth.

[17] Gebreweld A, Erkihun Y, Feleke DG, Hailu G, Fiseha T (2021). Thrombocytopenia as a diagnostic marker for malaria in patients with acute febrile illness. J Trop Med.

[18] Fried M, Muehlenbachs A, Duffy PE (2012). Diagnosing malaria in pregnancy: an update. Expert Rev Anti Infect Ther.

[19] Jairajpuri ZS, Rana S, Hassan MJ, Nabi F, Jetley S (2014). An analysis of hematological parameters as a diagnostic test for malaria in patients with acute febrile illness: an institutional experience. Oman Med J.

[20] Stanley M, Chippa V, Aeddula NR, Quintanilla Rodriguez BS, Adigun R (2024). Rhabdomyolysis. https://www.ncbi.nlm.nih.gov/books/NBK448168/.

[21] Castera MR, Borhade MB (2024). Fluid Management. https://www.ncbi.nlm.nih.gov/books/NBK532305/.

[22] Kuethe F, Pfeifer R, Rummler S, Bauer K, Kamvissi V, Pfister W (2009). Treatment of a patient with shock complicating severe falciparum malaria: a case report. Cases J.

[23] Zakama AK, Gaw SL (2019). Malaria in pregnancy: what the obstetric provider in nonendemic areas needs to know. Obstet Gynecol Surv.

[24] Boggild A, Brophy J, Charlebois P (2014). Summary of recommendations for the diagnosis and treatment of malaria by the Committee to Advise on Tropical Medicine and Travel (CATMAT). Can Commun Dis Rep.

[25] Peterson W, Tse B, Martin R, Fralick M, Sholzberg M (2021). Evaluating hemostatic thresholds for neuraxial anesthesia in adults with hemorrhagic disorders and tendencies: a scoping review. Res Pract Thromb Haemost.

[26] Harbell MW, Dumitrascu C, Bettini L, Yu S, Thiele CM, Koyyalamudi V (2021). Anesthetic considerations for patients on psychotropic drug therapies. Neurol Int.

[27] Kamel I, Ahmed MF, Sethi A (2022). Regional anesthesia for orthopedic procedures: what orthopedic surgeons need to know. World J Orthop.

[28] Ashken T, West S (2021). Regional anaesthesia in patients at risk of bleeding. BJA Educ.

[29] Shah S, Banh ET, Koury K, Bhatia G, Nandi R, Gulur P (2015). Pain management in pregnancy: multimodal approaches. Pain Res Treat.

[30] Queremel Milani DA, Davis DD (2024). Pain Management Medications. https://www.ncbi.nlm.nih.gov/books/NBK560692/.

[31] Cholette JM, Lerner NB (2011). Use of blood products. Pediatric Critical Care Study Guide.

[32] Sartelli M, Pagani L, Iannazzo S (2020). A proposal for a comprehensive approach to infections across the surgical pathway. World J Emerg Surg.

[33] Horn R, Hendrix JM, Kramer J (2024). Postoperative Pain Control. https://www.ncbi.nlm.nih.gov/books/NBK544298/.

[34] Habboush Y, Yarrarapu SNS, Guzman N (2024). Infection Control. https://www.ncbi.nlm.nih.gov/books/NBK519017/.

[35] Zanfini BA, Dell'Anna AM, Catarci S, Frassanito L, Vagnoni S, Draisci G (2016). Anesthetic management of urgent cesarean delivery in a parturient with acute malaria infection: a case report. Korean J Anesthesiol.

[36] Samanta S, Samanta S, Haldar R (2014). Emergency caesarean delivery in a patient with cerebral malaria-leptospira co infection: anaesthetic and critical care considerations. Indian J Anaesth.

[37] Bajwa SJ, Bajwa SK (2013). Anaesthetic challenges and management during pregnancy: strategies revisited. Anesth Essays Res.

[38] Addy JW, Bediako Y, Ndungu FM (2021). 10-year longitudinal study of malaria in children: insights into acquisition and maintenance of naturally acquired immunity. Wellcome Open Res.

